# Obesity Medications and Their Impact on Cardiovascular Health: A Narrative Review

**DOI:** 10.7759/cureus.71875

**Published:** 2024-10-19

**Authors:** Kazi N Islam, Rahib K Islam, Victoria T Tong, M Zaid Shami, Kaitlyn E Allen, Jared R Brodtmann, Jordan A Book

**Affiliations:** 1 Agricultural Research Development Program, Central State University, Wilberforce, USA; 2 School of Medicine, Louisiana State University (LSU) Health Sciences Center New Orleans, New Orleans, USA; 3 Internal Medicine, Aventura Hospital and Medical Center, Miami, USA

**Keywords:** cardiovascular health, glp-1 receptor agonists, naltrexone-bupropion, obesity, orlistat, phentermine-topiramate, setmelanotide, weight management

## Abstract

Obesity is a major global issue linked to cardiovascular diseases (CVDs). While lifestyle changes are the primary treatment, medications are often required for long-term weight management and reducing risk in patients with obesity. The cardiovascular effects of many obesity medications are still being studied. This review examines the cardiovascular impact of commonly prescribed obesity medications, focusing on their mechanisms, effectiveness, and safety. A review of the literature was conducted to evaluate the cardiovascular effects of these drugs, including their impact on major cardiovascular outcomes, cholesterol, blood pressure, and other heart-related factors. Some medications, like glucagon-like peptide-1 receptor agonists (GLP-1 RAs), show cardiovascular benefits, while others like orlistat have a lesser effect. Medications such as naltrexone-bupropion and phentermine-topiramate offer weight loss but still require further review for their cardiovascular safety. Data on setmelanotide's long-term effects are limited. Obesity medications differ in their effects on cardiovascular health, with some offering more consistent benefits. More studies are needed to fully understand their long-term risks and benefits, but combining medication with lifestyle changes remains key to improving both weight and heart health.

## Introduction and background

Obesity is a rapidly growing global health crisis, contributing significantly to morbidity and mortality worldwide [1]. According to the World Health Organization (WHO), the global prevalence of obesity has more than tripled since 1975, and as of the most recent estimates, over 650 million adults are considered obese, representing about 13% of the world’s adult population [2]. Obesity is clinically defined by a body mass index (BMI) of 30 kg/m² or greater, with BMI serving as a widely accepted metric to classify weight status. A BMI between 25 and 29.9 kg/m² is categorized as overweight, while a BMI of 30 kg/m² or higher indicates obesity [3].

The rise in obesity has not only imposed substantial burdens on healthcare systems but has also dramatically increased the risk of developing a wide array of comorbidities, particularly cardiovascular diseases (CVDs) [4]. Obesity mechanistically contributes to cardiovascular risk through several pathways, including the development of hypertension, dyslipidemia, insulin resistance, and systemic inflammation. These metabolic disturbances accelerate atherosclerosis, increase cardiac workload, and impair vascular function, collectively heightening the risk of coronary artery disease, stroke, and heart failure [5].

Given the high prevalence of both obesity and obesity-related CVDs, medical intervention is often necessary to manage body weight and reduce cardiovascular risk [6]. Several pharmacological agents have been developed to aid in weight reduction, each with distinct mechanisms of action and varying effects on cardiovascular health [7].

This paper reviews the cardiovascular implications of several major obesity medications, including glucagon-like peptide-1 receptor agonists (GLP-1 RAs), orlistat, naltrexone-bupropion, phentermine-topiramate, and setmelanotide. By examining these medications' benefits, mechanisms, and adverse cardiovascular effects, this review aims to provide clinicians with an updated understanding of how these pharmacotherapies impact cardiovascular health in obese patients.

## Review

Methods

This narrative review was conducted by performing a comprehensive literature search using the PubMed database to identify relevant studies on obesity pharmacotherapy and its cardiovascular implications. The search encompassed articles published up until [insert date], with no strict time limits applied. Search terms included combinations of keywords such as “obesity,” “cardiovascular health,” “pharmacotherapy,” “GLP-1 receptor agonists,” “orlistat,” “naltrexone-bupropion,” “phentermine-topiramate,” “setmelanotide,” and related terms for obesity medications.

Given the narrative nature of this review, no formal inclusion or exclusion criteria were strictly applied. Articles were selected based on their relevance to the topics of obesity pharmacotherapy, cardiovascular health, and the mechanisms of action of the drugs under discussion. Priority was given to original research, clinical trials, and large-scale meta-analyses, while review articles and expert opinions were included to provide additional context where necessary.

Data extraction was performed manually by the authors, focusing on the cardiovascular outcomes, mechanisms of action, and efficacy of obesity medications. No quantitative synthesis or meta-analysis was performed due to the nature of the narrative review. Instead, the key findings from the selected studies were synthesized to provide an updated overview of how obesity medications impact cardiovascular health.

Recent trends in obesity prevalence and associated cardiovascular risks

Obesity remains a growing public health challenge with significant implications for cardiovascular health. According to the World Health Organization (WHO), global obesity prevalence has risen steadily, now affecting approximately 43% of the global population [2]. In the United States, obesity has the highest prevalence rate, at 23.2% [2]. The rise in obesity is not limited to adults; childhood obesity is also on the rise, with implications for long-term cardiovascular risks.

Obesity is a well-established risk factor for cardiovascular diseases (CVD), including coronary heart disease, stroke, and heart failure. Recent studies further underscore the magnitude of this relationship. For example, a study by Alkhawam et al. found that out of 414 obese individuals with a body mass index (BMI) over 30, 332 (80%) had evidence of coronary artery disease (CAD) (p<0.001) [8]. In a population-based case-control study, individuals who were obese, characterized by having a BMI of over 30, were associated with an increased chance of having a stroke (odds ratio, 1.57, 95% C.I. = 1.28-1.94) [9].

The upward trend in obesity and its associated cardiovascular risks underscores the need for continued public health efforts targeting obesity prevention and management. Understanding the current data and emerging trends in obesity is critical for shaping interventions that can effectively mitigate the rising burden of cardiovascular disease.

GLP-1 agonists (semaglutide, liraglutide, tetatrutide, etc.)

Glucagon-like peptide-1 receptor agonists (GLP-1 RA), also known as incretin mimetics, represent a class of prescription medications used to treat hyperglycemia in patients with type 2 diabetes mellitus and promote weight loss in obese patients [10]. Due to its cardioprotective and neuroprotective effects, more research has been underway to determine the effect of GLP-1 RAs in reducing the risk of certain cancers, cardiovascular disease, and neurological diseases [11,12]. 

Mechanism of Action

Glucagon-like peptide-1 receptor agonists (GLP-1 RAs) bind to GLP-1 receptors and mimic the effects of GLP-1 in the pancreas, gastrointestinal system, and central nervous system. GLP-1 is a naturally occurring incretin hormone released by gut enteroendocrine L cells in response to the ingestion of nutrients, such as food [13]. GLP-1 RAs potentiate glucose-mediated depolarization of voltage-gated calcium channels, resulting in an influx of calcium that triggers vesicular exocytosis and secretion of GLP-1 into the circulation [14]. GLP-1 RAs can only potentiate the downstream actions of elevated glucose concentrations in the pancreas and therefore have minimal activity in the absence of elevated blood glucose levels [15]. GLP-1 binds to beta cells in the pancreas to stimulate glucose-dependent insulin release and alpha cells to reduce glucagon release [16]. Through its actions on central receptors in the hypothalamus, GLP-1 reduces appetite and increases satiety, independent of its effects on the stomach [17]. GLP-1 acts on central and peripheral receptors to decrease gastric acid secretion, gastric emptying, and gut motility, which contribute to the feeling of fullness and satiety [18,19].

Beneficial Effects

By regulating insulin secretion and improving glycemic control, GLP-1 RAs may reduce postprandial hyperglycemia and body weight [20]. GLP-1 has potent effects on cholesterol metabolism and has been found to significantly reduce serum levels of low-density lipoproteins (LDL) cholesterol, total cholesterol, and triglycerides compared to control patients [21]. GLP-1 RAs may slow the progression of chronic kidney disease and reduce the need for dialysis in diabetic patients [22]. GLP-1 RAs have also been found to reduce inflammation, systolic and diastolic blood pressure, and hepatic injury [23,24].

Adverse Effects

GLP-1 RAs cause adverse effects related to their mechanism in the gastrointestinal tract. These adverse effects may include dose-dependent nausea, vomiting, diarrhea, constipation, abdominal pain, and dyspepsia [25]. Although there are some reports of GLP-1 RAs inducing hypoglycemia, there is no clear evidence that this effect is mediated solely by GLP-1 Ras [26]. There is a small risk of GLP-1 RAs inducing mild hypoglycemia when taken in combination with other drugs, such as sulfonylureas, or when other risk factors are present [27]. Rarely, GLP-1 RAs may cause severe symptoms such as pancreatitis, gastroparesis, bowel obstruction, or biliary disease [28-31].

Effects on Cardiovascular Health

GLP-1 RAs have been associated with a reduction in the rate of major adverse cardiovascular events by 12% (HR 0·88, 95% CI 0·82-0·94; p<0·001) [32]. GLP-1 RAs bind to GLP-1 receptors expressed in cardiomyocytes and vascular endothelial cells to cause beneficial effects on cardiovascular health, such as reductions in cardiovascular mortality (OR: 0.88; 95% CI: 0.80-0.96; p < 0.01; I2 = 14) and odds of stroke (OR: 0.84; 95% CI: 0.76-0.93; p < 0.01; I2 = 0%) in diabetic patients [33]. GLP-1 agonists lower plasma lipid levels and blood pressure, which reduces the risks associated with hypertension and cardiovascular remodeling. The cholesterol-lowering effects of GLP-1 RAs prevent the formation of atherosclerotic plaques, contributing to reductions in atherothrombotic events and atherosclerotic cardiovascular disease [34,35].

GLP-1 receptor agonists have been extensively studied for their long-term cardiovascular effects, with multiple large-scale cardiovascular outcome trials (CVOTs) supporting their cardioprotective benefits. The LEADER trial followed over 9340 patients for a median of 3.8 years. It demonstrated a significant 13% relative risk reduction in major adverse cardiovascular events (MACE) (composite of cardiovascular death, non-fatal myocardial infarction, and non-fatal stroke) with liraglutide compared to placebo (HR 0.87, 95% CI 0.78-0.97, P = 0.01). The trial also showed a 22% reduction in cardiovascular death and a 15% reduction in all-cause mortality [36].

Similarly, the SUSTAIN-6 trial assessed semaglutide in a population with high cardiovascular risk and showed a 26% reduction in MACE (HR 0.74, 95% CI 0.58-0.95), with the added benefit of a significant reduction in stroke risk (39% relative risk reduction, HR 0.61, 95% CI 0.38-0.99) over a median follow-up period of 2.1 years [37].

The REWIND trial focused on a more general population with type 2 diabetes and found that dulaglutide was associated with a 12% reduction in MACE (HR 0.88, 95% CI 0.79-0.99) over a median follow-up of 5.4 years. Notably, the trial demonstrated that these benefits extend to patients with a lower baseline cardiovascular risk than those in the LEADER and SUSTAIN-6 trials [38].

In addition to these reductions in cardiovascular events, GLP-1 receptor agonists are known to have favorable effects on blood pressure, with an average reduction of 2-3 mmHg in systolic blood pressure and on lipid profiles, particularly through lowering LDL cholesterol and triglyceride levels. These benefits are thought to stem from their direct effects on vascular function and inflammation, as well as indirect effects mediated through weight loss and glycemic control [35].

Given the robust evidence from multiple long-term trials, GLP-1 receptor agonists not only offer substantial benefits in managing glucose levels and promoting weight loss but also significantly reduce the risk of cardiovascular events. These outcomes position GLP-1 receptor agonists as a valuable therapeutic option for patients with obesity and concomitant cardiovascular risk factors. However, further long-term studies are needed to fully understand the impact of these drugs on non-diabetic populations and to explore any potential class-specific differences in cardiovascular outcomes.

Orlistat

Orlistat (tetrahydrolipstatin) is a lipase inhibitor indicated for weight loss and management in overweight and obese adults [39]. Due to its beneficial effects on cardiovascular health, orlistat is also prescribed off-label for treating obesity in patients with heart failure and lowering serum triglycerides in children with type 1 hyperlipoproteinemia [40,41].

Mechanism of Action

Lipases are digestive enzymes responsible for hydrolyzing triglycerides into free fatty acids and monoglycerides that can be readily absorbed into the body for energy and fat stores [42]. Orlistat covalently binds the serine residues located on the active site of gastric and pancreatic lipases, forming a stable complex that prevents them from metabolizing dietary fat [39]. Dietary fats that cannot be absorbed in the intestines are excreted into the stool, facilitating weight loss.

Beneficial Effects

Orlistat’s weight-reducing effects are clinically significant compared to lifestyle and diet modifications alone [43]. At recommended therapeutic doses, orlistat can minimize dietary fat absorption by 30%, resulting in reductions in body weight, BMI, and waist circumference [39,44,45]. The weight loss associated with orlistat may reduce the risk of obese patients developing impaired glucose tolerance and type 2 diabetes [46]. Orlistat can improve lipid profiles by lowering total cholesterol (-11.9% vs. -4.0%; P<0.001), LDL cholesterol (-17.6 vs. -7.6%; P<0.001), and triglycerides. A 2001 study revealed that orlistat has a direct cholesterol-lowering effect that is independent of weight reduction (P<0.001) [47].

Adverse Effects

Orlistat is generally well tolerated with a safety profile comparable to placebo in all organ systems, with the exception of a higher incidence of gastrointestinal events [47]. The most common adverse effects reported for orlistat are categorized as gastrointestinal disorders, metabolism and nutrition disorders, renal and urinary disorders, musculoskeletal and connective tissue disorders, and hepatobiliary disorders [48]. Due to its primary actions in the gastrointestinal system, most of orlistat’s adverse effects are associated with malabsorption of fats and fat-soluble vitamins, specifically abdominal pain, diarrhea, steatorrhea, and fecal spotting [49]. Other symptoms include body aches, and nausea, Rarely, orlistat may cause severe symptoms such as hepatotoxicity, cholelithiasis, or pancreatitis [50-52].

Effects on Cardiovascular Health

A propensity-score-matched study performed in 2022 found that orlistat was associated with lower rates of overall major adverse cardiovascular events. Patients who took orlistat experienced lower rates of myocardial infarction (HR 0.77; 95% CI 0.66-0.88, P < 0.001), ischemic stroke (HR 0.68; 95% CI 0.56 to −0.84, P < 0.001), and new-onset heart failure (HR 0.79; 95% CI 0.67-0.94, P = 0.007) [53]. These findings suggest that orlistat may confer cardiovascular benefits beyond weight reduction, particularly by modulating lipid metabolism and reducing inflammation. However, the study acknowledged limitations, including its observational design and the need for randomized controlled trials (RCTs) with a primary cardiovascular endpoint [54]. In animal models, orlistat has shown the potential to mitigate obesity-related myocardial damage by reducing oxidative stress, inflammation, and apoptosis. A study in obese rats found that orlistat alleviated myocardial damage and atherosclerosis progression through improved lipid metabolism and reductions in pro-inflammatory markers. Although promising, these preclinical findings require further validation in human studies [55,56].

Although orlistat is primarily known for its role in reducing dietary fat absorption and promoting weight loss, its long-term cardiovascular effects have been investigated in several studies, with mixed results. Orlistat has demonstrated efficacy in improving lipid profiles, particularly by lowering total cholesterol, LDL cholesterol, and triglycerides. In the XENDOS trial, which followed over 3,000 obese patients for four years, orlistat not only promoted significant weight loss but also reduced the incidence of type 2 diabetes by 37% (P < 0.001). However, despite improvements in metabolic markers, this trial did not find a statistically significant reduction in major adverse cardiovascular events [43].

While orlistat has demonstrated improvements in lipid profiles and may reduce the risk of cardiovascular events such as myocardial infarction and stroke, the long-term cardiovascular benefits remain inconclusive due to a lack of large-scale, long-term RCTs specifically designed to assess cardiovascular outcomes. Future studies should establish whether orlistat's cardiovascular benefits extend beyond its effects on weight loss and lipid profiles. In clinical practice, orlistat may be considered a safe option for patients seeking weight loss with potential additional benefits for cardiovascular health, particularly in patients with dyslipidemia or those at risk of metabolic syndrome.

Naltrexone-Bupropion

Naltrexone-bupropion was approved by the FDA in 2014 for chronic weight management in adults with a BMI of 30 kg/m^2^ or greater or with a BMI of 27 kg/m^2^ or greater coupled with at least one weight-related comorbid condition. Although this combination drug may not be the first option for all patients for weight management, it can be especially helpful for those also attempting to quit smoking or with concurrent depression [57]. The theorized mechanism of action for weight loss is primarily through bupropion, which is a stimulator of proopiomelanocortin (POMC) production. Alpha-MSH, a downstream product of POMC, stimulates the melanocortin-4 receptor (MC4R), leading to increased energy expenditure, decreased appetite, and consequent weight loss. However, the increased levels of beta-endorphin, an endogenous opioid product also derived from POMC, provide negative feedback on this system via the mu-opioid receptor. As an opioid antagonist, naltrexone is paired with bupropion to block the inhibitory effect of beta-endorphin and enhance the desired weight loss [57,58].

A main benefit of naltrexone-bupropion is significant weight loss, especially when combined with behavioral modification. As seen in the randomized, placebo-controlled trials by Wadden et al. and Helseth et al., naltrexone 32 mg/day plus bupropion 360 mg/day (NB32) paired with diet and exercise resulted in more significant weight loss than placebo and behavioral modifications alone [59,60]. In two other trials studying pharmacotherapy alone, weight loss was also significantly greater with naltrexone-bupropion than placebo [61,62]. With weight loss, studies typically show decreased risk for cardiovascular events; however, the effect of NB32 on cardiovascular metrics has been uncertain [63]. Some metrics, such as high-density lipoprotein (HDL) cholesterol and triglycerides, were significantly improved with NB32, as seen in Greenway et al., Apovian et al., and Wadden et al., but the participant characteristics in these studies excluded patients with existing significant cardiovascular disease [59,61,62]. Studies have also shown that NB32 might attenuate improvements in heart rate and blood pressure that significant weight loss would normally produce [59,61]. Unfortunately, the large-scale randomized clinical trial that was intended to investigate cardiovascular safety for NB32 was stopped prematurely due to the sponsor releasing confidential interim study results. Preliminary results suggested that fewer major adverse cardiovascular events (MACE) were reported in the treatment group, but the study did not generate enough statistical power to make an official claim [64]. In a 2021 meta-analysis by Sposito et al., the cardiovascular safety of naltrexone-bupropion for the treatment of obesity, smoking cessation, and constipation was investigated. This analysis found no significant association between this combination drug or its individual constituents and MACE (naltrexone-bupropion: odds ratio = 0.97 (95% CI 0.75-1.24), p = 0.79) [65].

The side effects and interactions associated with naltrexone-bupropion are essentially the sum of its parts. As an opioid antagonist, naltrexone can have several drug interactions with other medications utilizing the mu-opioid receptor, such as cough suppressants, opioid analgesics, or anti-diarrheal agents [58]. Bupropion is contraindicated in patients utilizing monoamine oxidase inhibitors due to the risk of hypertension. Because bupropion is a strong inhibitor of cytochrome P4502D6, the dosages of other medications metabolized by this isozyme must be titrated accordingly. This combination drug is also contraindicated in patients with existing seizure disorders, and extra caution should be taken for patients taking antipsychotics, antidepressants, or theophylline, as bupropion can lower the seizure threshold [57,58]. Lastly, in several randomized controlled trials comparing naltrexone-bupropion to placebo, common adverse effects reported were nausea, headache, and gastrointestinal distress [57-62].

Effects on Cardiovascular Health

The combination of naltrexone and bupropion (NB32) is approved for chronic weight management, primarily through its effects on appetite suppression and energy expenditure. However, its cardiovascular safety has been the subject of ongoing research, with mixed findings from clinical trials.

The CONTRAVE Obesity Research (COR) program, a series of Phase 3 clinical trials, demonstrated that NB32 led to significant weight loss compared to placebo, with reductions in waist circumference, improved glycemic control, and modest improvements in lipid profiles (HDL cholesterol and triglycerides). Despite these metabolic benefits, concerns about its impact on heart rate and blood pressure have been raised, which may offset some of its cardiovascular advantages [62].

In a 2021 meta-analysis by Sposito et al., the cardiovascular effects of NB32 were further investigated. This analysis, which pooled data from various trials, found no significant association between NB32 use and an increased risk of MACE. However, the meta-analysis highlighted that the populations included in these studies often excluded individuals with pre-existing cardiovascular conditions, making it difficult to assess the drug’s safety in high-risk populations [65].

While initial trials indicate that NB32 is unlikely to increase the risk of major cardiovascular events in the general population, the long-term cardiovascular safety of this combination therapy remains underexplored due to the early termination of key studies. Further research, particularly in populations with established cardiovascular disease, is necessary to provide more robust data. Clinicians should consider the potential benefits of weight loss and metabolic improvements against the possible risks of increased heart rate and blood pressure when prescribing NB32 for patients with cardiovascular comorbidities.

Phentermine-Topiramate

The phentermine-topiramate extended-release capsule was approved by the FDA in 2012 as a supplemental weight loss therapy to a reduced-calorie diet and physical exercise for adult patients with a BMI greater than 30 kg/m^2^ or patients with a BMI equal to or greater than 27 kg/m^2^ with at least one comorbid condition, including hypertension, hyperlipidemia, and type 2 diabetes mellitus. It is currently sold under the brand name Qsymia. An additional supplemental indication was added by the FDA for pediatric patients 12 years of age or older in 2022 who are clinically defined as obese, or rather equal to or greater than the 95th percentile of BMI for their respective age and sex. Again, as in adults, the recommendation is to use this drug as a supplement to diet and exercise in pediatric patients [66,67].

Mechanism of Action

Phentermine exerts its effects of weight loss via sympathetic stimulation. It acts primarily as a central appetite suppressant, very similar to amphetamine. In fact, it is a derivative of amphetamine with an alpha-methyl substitution present at the phenylethylamine side chain. Importantly, it is a schedule IV-controlled substance on its own due to its addictive properties. However, lower doses are being tolerated as an effective weight loss adjunct, largely due to its decreased CNS side effects as a result of its biochemical structure [67,68].

Topiramate is a staple drug in treating epilepsy and for migraine prophylaxis, but its effects on weight loss are less documented in the medical community. It acts as an inhibitor of voltage-gated sodium channels as well as possibly voltage-gated calcium channels but has many other possible substrates. In the avenue of weight loss, topiramate is believed to express its effects via resensitizing multiple cell lines to insulin via its antagonistic properties at ion channels. There has also been evidence of topiramate having an inhibitory effect on neuropeptide y, an important positive regulator of hunger at the hypothalamus [67,68].

Efficacy

The efficacy of phentermine/topiramate (PHEN/TPM) delivered as one drug as opposed to prescribing each drug separately was tested in a 28-week randomized control trial in 2013 by Aronne et al. Subjects were randomized to seven control groups: placebo, phentermine 7.5 mg, phentermine 15 mg, topiramate ER 46 mg, topiramate ER 92 mg, PHEN/TPM ER 7.5/46 mg, or PHEN/TPM ER 15/92 mg. It was found that PHEN/TPM ER 7.5/46 (-8.5%) and 15/92 (-9.2%) achieved greater percentages of weight loss versus placebo (-1.7%; P < 0.0001) and the monotherapy formulations of the drugs (P < 0.05) [69]. The longitudinal efficacy of the drug combo related to weight loss was best explored in the EQUIP, SEQUEL, and CONQUER trials. The EQUIP trial was the first to be performed in 2012 and explored obese adult men and women with BMI greater than 35. Three groups were established: one placebo, one low dose of PHEN/TPM, and one high dose of PHEN/TPM, and were observed for 56 weeks. The low-dose group lost 5.1% of their starting body weight, and the high-dose group lost 10.9% of their starting body weight, compared to 1.6% for the placebo group. Around 44.9% of low-dose participants lost 5% or more of their body weight, as well as 66.7% of the high-dose participants [70]. This paved the way for the SEQUEL and CONQUER trials to follow, which altered the low dose to a slightly higher but still lower dose than the high dose. The trends found in the EQUIP trial were similar to those found in its successors, which followed its participants for a longer period of time of four years [70-72].

Effects on Cardiovascular Health

Across multiple randomized controlled trials, phentermine-topiramate consistently showed significant decreases in body weight compared to placebos. Improvements in dose and length of treatment have been elucidated over these trials. Meanwhile, the evidence of improvements in metabolic values that can accurately predict cardiovascular health was less consistent. Only when patients were on the drug combo for four years were some cardiometabolic markers shown to be significantly improved, in the SEQUEL trial. The researchers did state however that the need for antihypertensive therapy decreased with phentermine-topiramate use compared to placebo, thereby reducing their polypharmacy and treatment costs [71,73,74]. In years after the adult trials, adolescent trials showed similar data for positive weight loss but inconclusive cardiovascular benefits [66]. An additional trial explored the cardiovascular comorbidity of type 2 diabetes mellitus, and it was found after 106 weeks of low-dose and high-dose PHEN/TPM that the drug combo produced significant reductions of 70.5% in the low-dose group and 78.7% in the high-dose group in the annualized incidence rate of type 2 diabetes mellitus [75].

Adverse Effects

Concerns for side effects of this drug exist, however. The most common side effects experienced across trials were dry mouth and paresthesias. However, in the context of cardiovascular health, although minimal positive cardiovascular effects have been observed, doubt remains in phentermine-topiramate’s use in high-risk cardiovascular patients, especially those with coronary artery disease or uncontrolled hypertension, in hyperthyroid patients, and glaucoma patients due to phentermine’s sympathomimetic effects on heart rate, blood pressure, and intraocular pressure. This could correlate to poor outcomes for these cross-sections of the patients in need of weight loss medical therapy. In addition, contraindications also exist for pregnant patients, as topiramate has been indicated as a risk factor for cleft lip and cleft palate malformations in newborns [67,68,70-72,74].

Setmelatonide

Setmelatonide was approved by the FDA as a supplemental adjunct to a calorie-deficit diet and physical exercise for weight loss in 2022. The drug is administered as a once-daily subcutaneous injection. The drug is specifically indicated for patients with decreased POMC, PCSK1, or leptin receptor (LEPR) or patients diagnosed with a rare genetic syndrome called Bardet-Biedl syndrome that presents with cognitive delays, short stature, and obesity in the pediatric population [76,77].

Mechanism of Action

Setmelatonide works by activating the melanocortin-4 receptor (MC-4), a central nervous system receptor implicated in the hypothalamic regulation of satiety and overall energy usage. This explains its prominent effectiveness in the aforementioned patient populations, as stimulating this receptor directly helps to reestablish a deficient level of satiety with meals and decreased caloric expenditure seen in all four cross sections of obese patients needing weight loss [78,79].

Efficacy

The benefits of this therapy for weight loss in these patients have been overwhelmingly positive. In a study by Clément et al. in 2020, 80% of patients with POMC deficiency and 45% of patients with LEPR deficiency achieved a goal of 10% or more weight loss of their starting weight in the clinical trial [76]. Similarly, MCR4-deficient patients were evaluated in a separate study, and setmelanotide again showed significant weight loss benefits in 2017 compared to placebo [80]. Most recently, studies completed in 2020 and 2022 evaluated the drug’s effectiveness in patients with Bardet-Biedl syndrome, and the agonist once again proved its worth with significant decreases in body weight compared to placebo [77,81]. Although these studies did not primarily evaluate cardiovascular outcomes, the significant weight reduction and improvements in lipid profiles imply that setmelanotide may have a favorable effect on cardiovascular health in these patients. However, long-term follow-up studies are needed to assess whether these metabolic improvements translate into reductions in cardiovascular events like myocardial infarction, stroke, or heart failure.

Effects on Cardiovascular Health

In terms of the drug’s cardiovascular protective effects, research on this front is limited due to the majority of populations that show indications for the drug are of pediatric age and therefore are not at as high of a risk of significant adverse cardiovascular events. However, Clément’s study in 2020 did show significantly positive effects for HDL and LDL levels in both POMC and LEPR deficiency, suggesting potential cardiovascular benefits through improved metabolic parameters [76]. The drug did not cause any reported adverse cardiovascular events in any of the trials leading to its FDA approval in 2022 [76,77,80,81].

Adverse Effects

Adverse effects of the drug have come to light, most commonly in the form of hyperpigmentation as a result of setmelatonide’s stimulation of other melanocortin receptors, particularly melanocortin-1 receptors in the skin, across the body. Some studies reported symptoms of nausea and vomiting, while others noted erythema surrounding the injection site since the drug is delivered subcutaneously. Other adverse effects documented include depression and suicidal ideation, decreases in sexual arousal and interest, and anaphylaxis. The only FDA contraindication to the drug is for the history of anaphylaxis to setmelanotide [76,77,79].

Comparison of key obesity medications: mechanisms, efficacy, cardiovascular effects, and adverse events is detailed in Appendix 1.

Emerging obesity medications and their cardiovascular impacts

As research in obesity pharmacotherapy advances, newer medications are emerging with promising efficacy in weight management and potential cardiovascular benefits. Among these are tirzepatide and cagrilintide, both of which are currently under investigation for their dual role in glycemic control and weight reduction. Understanding their cardiovascular implications is crucial, as obesity remains a major contributor to cardiovascular morbidity.

Tirzepatide

Tirzepatide is a novel dual glucose-dependent insulinotropic polypeptide (GIP) and glucagon-like peptide-1 (GLP-1) receptor agonist. It has been extensively studied for its ability to promote weight loss and improve glycemic control in patients with type 2 diabetes. In the SURPASS-5 trial, tirzepatide was evaluated in combination with insulin glargine for its efficacy in patients with type 2 diabetes and inadequate glycemic control [82].

This randomized, phase 3 clinical trial included 475 adults who received tirzepatide at doses of 5 mg, 10 mg, or 15 mg, or a placebo over 40 weeks. Tirzepatide led to significant reductions in glycated hemoglobin A1c (HbA1c), with decreases of -2.21% for the 5 mg dose, -2.40% for the 10 mg dose, and -2.34% for the 15 mg dose, compared to -0.86% with placebo (p < 0.001 for all doses). Additionally, body weight decreased by -5.4 kg, -7.5 kg, and -8.8 kg for the 5 mg, 10 mg, and 15 mg doses, respectively, while patients in the placebo group gained 1.6 kg (p < 0.001 for all doses). Furthermore, the percentage of patients achieving HbA1c levels below 7% was significantly higher in the tirzepatide groups, ranging from 85% to 90%, compared to 34% in the placebo group (p<0.001 for all comparisons) [83].

Although the primary focus of the SURPASS-5 trial was on glycemic control, the substantial weight loss observed suggests potential cardiovascular benefits, as weight reduction is closely linked to improved cardiovascular outcomes. Further studies are required to confirm reductions in MACE.

Cagrilintide

Cagrilintide is another emerging medication that acts as an amylin analog, working in conjunction with GLP-1 receptor agonists to enhance weight loss through appetite suppression and delayed gastric emptying [84]. While cagrilintide has shown promising results in weight reduction, with clinical trials demonstrating significant reductions in body weight when combined with semaglutide, its cardiovascular effects remain an area of active investigation.

In a thorough QT study, cagrilintide administered at escalating doses of up to 4.5 mg did not result in clinically relevant QT prolongation compared to placebo, demonstrating no increased risk of ventricular arrhythmias. This confirms its cardiac safety in terms of electrical repolarization [85].

In a separate randomized, multicenter, double-blind phase 2 trial, cagrilintide (0.3-4.5 mg) led to significant weight reductions in participants with a body mass index (BMI) ≥30 kg/m² or ≥27 kg/m² with hypertension or dyslipidemia. The greatest weight loss occurred with the 4.5 mg dose, with a mean reduction of 10.8% (11.5 kg) compared to 9.0% (9.6 kg) with liraglutide 3.0 mg and 3.0% (3.3 kg) with placebo (p < 0.001). The most common adverse events were gastrointestinal-related, including nausea and constipation [86].

These findings position cagrilintide as a promising agent for obesity treatment, with both effective weight loss and a favorable cardiac safety profile.

Future directions in obesity pharmacotherapy and cardiovascular health

As obesity continues to drive global morbidity and mortality, future research in obesity pharmacotherapy is poised to explore novel approaches that not only enhance weight loss but also improve cardiovascular outcomes. Current medications such as GLP-1 receptor agonists and dual GIP/GLP-1 receptor agonists like tirzepatide show promise, particularly in their ability to reduce major adverse cardiovascular events (MACE) alongside weight loss. Moving forward, there is increasing interest in the development of multi-target drugs that simultaneously address metabolic pathways contributing to both obesity and cardiovascular risk.

Personalized medicine is also expected to play a more prominent role, with a focus on tailoring pharmacotherapy based on an individual's genetic, metabolic, and cardiovascular profiles. This approach could help optimize the efficacy of treatments while minimizing adverse effects. Additionally, long-term studies will be essential in assessing the sustainability of weight loss and the durability of cardiovascular benefits in diverse populations, including those without diabetes or preexisting cardiovascular disease.

Furthermore, the combination of pharmacotherapy with advanced technologies, such as digital health tools and remote monitoring, may offer more comprehensive strategies for managing obesity and its cardiovascular consequences. Integrating these emerging therapies with lifestyle interventions will remain a critical challenge, but one that holds significant potential to mitigate the global burden of obesity and its associated cardiovascular risks.

## Conclusions

Obesity medications have emerged as essential tools in the management of obesity, especially when lifestyle interventions alone are insufficient to achieve significant weight loss. While medicines like GLP-1 RAs, orlistat, and phentermine-topiramate offer clear benefits in terms of weight reduction and improved metabolic parameters, their long-term effects on cardiovascular health require further investigation. Some medications have shown promise in reducing major adverse cardiovascular events (MACE), while others need more robust clinical trials to establish their cardiovascular safety profiles.

Future research should focus on evaluating the comparative cardiovascular effects of obesity medications and identifying patient subgroups that may benefit the most. Additionally, exploring the combination of pharmacological treatments with lifestyle changes remains a critical avenue for maximizing both weight loss and cardiovascular health outcomes. Developing novel therapeutic strategies that effectively balance weight management and cardiovascular risk reduction will be paramount as our understanding of obesity-related pathophysiology deepens.

## Appendices

Appendix 1

**Table 1:** Comparison of key obesity medications: mechanisms, efficacy, cardiovascular effects, and adverse events MACE: major adverse cardiovascular events; POMC: proopiomelanocortin; LEPR: leptin receptor; T2DM: type 2 diabetes mellitus; GI: gastrointestinal; MI: myocardial infarction

Medication	Mechanism of Action	Efficacy	Cardiovascular Effects	Adverse Effects
GLP-1 Receptor Agonists	Increases insulin secretion, reduces glucagon release, slows gastric emptying, and reduces appetite.	Significant weight loss and glycemic control. Studies show 10-13% weight reduction (e.g., LEADER, SUSTAIN-6 trials).	Reduces MACE by 12% (HR 0.88, 95% CI 0.82–0.94, p<0.001); improves lipid profiles, and blood pressure [32].	GI-related: nausea, vomiting, diarrhea; potential for hypoglycemia in combination with sulfonylureas.
Orlistat	Inhibits gastric and pancreatic lipases, reducing fat absorption.	30% reduction in fat absorption, modest weight loss (~3-5 kg); improves cholesterol levels.	Shown to lower MI risk (HR 0.77, 95% CI 0.66–0.88, p<0.001) and ischemic stroke risk (HR 0.68, 95% CI 0.56–0.84, p<0.001) [53].	GI disturbances: oily stools, fecal incontinence, fat-soluble vitamin deficiency.
Naltrexone-Bupropion	Bupropion activates POMC neurons, suppressing appetite; naltrexone blocks negative feedback.	Significant weight loss when combined with behavioral changes; 5-10% weight loss in clinical trials.	No significant increase in MACE (Odds Ratio = 0.97, 95% CI 0.75–1.24, p=0.79); potential adverse effects on blood pressure, and heart rate [65].	Nausea, headaches, GI distress, increased blood pressure, seizure risk in predisposed individuals.
Phentermine-Topiramate	Phentermine is a sympathomimetic agent suppressing appetite; topiramate affects multiple pathways, including appetite regulation.	8-10% weight loss in trials (e.g., EQUIP, CONQUER).	Shown to decrease antihypertensive therapy use; and potential improvements in T2DM outcomes [70].	Dry mouth, paresthesias, contraindicated in patients with uncontrolled hypertension or cardiovascular disease, pregnancy concerns (topiramate).
Setmelanotide	Activates the melanocortin-4 receptor, regulating satiety and energy expenditure.	Effective in rare genetic obesity disorders (10-20% weight loss in POMC/LEPR deficient patients).	Limited data; improves lipid profiles in specific populations; no major adverse cardiovascular effects observed in trials.	Hyperpigmentation, injection site reactions, nausea, depression, and suicidal ideation in some cases.
